# Association between anxiety, obesity and periodontal disease in smokers and non-smokers: A cross-sectional study

**DOI:** 10.15171/joddd.2016.037

**Published:** 2016-12-21

**Authors:** Abhay P. Kolte, Rajashri A. Kolte, Vrushali N. Lathiya

**Affiliations:** ^1^Department of Periodontics & Implantology, VSPM Dental College and Research Centre, Nagpur, India

**Keywords:** Anxiety, obesity, periodontal disease, risk factors

## Abstract

***Background.*** Psychological stress is known to be a relevant risk factor for many inflammatory conditions, including periodontal disease. A few studies have probed the relationship between obesity and periodontal disease. Therefore this cross-sectional study was aimed to examine the relationship between psychological stress and obesity and periodontal disease in smokers and non-smokers.

***Methods.*** The participants included 90 patients, equally divided into three groups of non-smokers and periodontally healthy, non-smokers and smokers with untreated moderate-to-severe chronic periodontitis. Socioeconomic data, psychosocial measurements, physical parameters and clinical findings of PPD, CAL, PI and GI were recorded.

***Results.*** The clinical parameters were assessed for three groups in three different anxiety levels of mild, moderate and severe. Intra-group comparison of PPD and CAL in the three anxiety levels showed increased periodontal destruction with an increase in anxiety levels, the results being statistically highly significant for PPD differences in smokers (P < 0.0001). The mean differences in PPD and CAL in severe anxiety levels between smokers and non-smokers were 0.68 mm and 0.70 mm and both the findings were statistically significant. The mean PPD and CAL in smoker and non-smoker groups in obese patients was higher as compared to non-obese patients and the differences were highly significant (P < 0.001).

***Conclusion.*** The results of our study indicated a positive and strong correlation between anxiety, obesity and periodontal disease in smokers and non-smokers. Smoking appears to further attenuate this association.

## Introduction

Periodontal diseases are one of the frequently observed inflammatory conditions affecting the periodontium and have been thought to be of multifactorial origin. Analysis of local, systemic and microbial factors of individuals affected with periodontal diseases indicate the role of multiple risk factors as robustly associated with the disease.^1^ These risk factors which have been currently understood to be established include advancing age, gender, smoking, plaque microorganisms and systemic diseases such as diabetes mellitus.^2,3^ However, a remarkable ratio of variation in affliction with periodontal diseases defy explanation, taking only these risk factors into account.^4^

Stress is thought to be another important risk factor for many inflammatory diseases, including periodontal diseases. It is observed that psychological stress regulates lifestyle features, leading patients to disregard oral hygiene, inducing smoking, behavioral modifications with consequential adverse effects on the gingival health, and at times, increased severity of periodontal diseases. Psychological and physical afflictions such as depression and anxiety influence the host defense mechanisms by exerting an immunosuppressive effect which ultimately increases the risk of disease. The association of periodontal infection such as acute necrotizing ulcerative gingivitis (ANUG) and susceptible psychological factors is well recognized. Preliminary investigations have laid stress on the consequence of psychological interruptions on initiation and progression of periodontal diseases.^5,6^

The relationship between smoking intensity and body weight has been evaluated in many investigations and obesity along with increased body weight and obesity levels have been found amongst the most intense smokers^7,8^ Also the literature indicates presence of preliminary positive evidence between obesity and periodontal disease.^9,10^

According to proposed mechanisms, obesity exerts influences on the host immunity and this interrelation is supposed to be linked to secretion of adipokines, including leptin. It has been evinced that human leptin is existent within the gingiva; however, this concentration decreases with an increase in severity of periodontal disease.^11^ Visceral adipose tissue is known to secrete adipokines which include tumor necrosis factor α that might also lead to further destruction of periodontal tissues, increasing the severity of the condition.

The above aspects suggest the possibility of a close correlation between anxiety, obesity and periodontal disease, but the evidence for such an association is scant. Therefore this cross-sectional study was planned to explore the relationship between psychological factors, obesity and periodontal disease in smokers and non-smokers.

## Methods

The study population comprised of 90 patients (69 males and 21 females) above 30 years of age, who attended the Department of Periodontics & Implantology, of our institute from August 2014 to July 2015. The patients were equally distributed into three groups as follows:

Group I: Thirty non-smokers and periodontally healthy patients

Group II: Thirty smokers with untreated moderate-to-severe chronic periodontitis affecting

more than 30% of the teeth, with periodontal attachment loss of ≥5 mm.

Group III: Thirty non-smokers with untreated moderate-to-severe chronic periodontitis affecting more than 30% of the teeth, with periodontal attachment loss of ≥5 mm.

Patients were excluded if they presented with systemic conditions associated with periodontal disease, history of periodontal treatment, self-reported psychiatric disorders or psychotic medications.

The Institutional Ethics Committee of VSPM Dental College & Research Centre approved the study protocol which complied with the Helsinki Declaration. The study details were explained to the participants individually and patients who volunteered to participate signed an informed consent form before being included.

### Socioeconomic data and clinical parameters

A detailed case history was recorded, which included socioeconomic data such as age, gender, educational level, status of employment, family earnings, level of education, family income and smoking history with the number of cigarettes smoked per day. Smoking exposure of the participant was reflected in terms of the number of cigarette consumption and duration. Only individuals in whom a distinct diagnosis of smoking could be established were included in the study. A standard questionnaire was used to generate information about smoking history.

The clinical periodontal parameters of gingival index (Loe & Silness),^12^ plaque index (Silness & Loe, 1964),^13^ probing pocket depth (PPD) and clinical attachment levels (CAL) were recorded along with physical examination parameters of height, weight and body mass index. The clinical periodontal parameters were recorded by one specialist (VL), while the other specialist (AK) recorded all the physical examination parameters. Body mass index (BMI) was computed as the body weight/height^2^ (kg/m^2^). Four BMI categories were defined using World Health Organization (WHO) criteria: Underweight (BMI ≤ 18.5 kg/m^2^), normal weight (BMI 18.5 to 24.9 kg/m^2^), overweight (BMI 25 to 29.9 kg/m^2^) and obese (BMI ≥ 30 kg/m^2^).

### Psychosocial measurements

The psychological measures to assess anxiety were made using Hamilton Anxiety Rating Scale (HAM-A).^14^The HAM-A is one of the first rating scales evolved to quantify the severity of anxiety manifestations and is commonly used even today in both clinical and research settings. The scale contemplates the use of 14 points, each identified by a series of traits which measure both psychic anxiety (mental agitation and psychological distress) and somatic anxiety (physical complaints related to anxiety). The reported levels of inter-rater reliability for the scale appears to be acceptable.^15^Each point is referenced on a scale of 0 (not present) to 4 (severe), with a total score range of 0‒56, where <17 indicates mild severity, 18‒24 indicates mild to moderate severity and 25‒30 moderate to severe anxiety.

### Statistical analysis

Continuous variables like age, PPD, CAL, PI, GI and BMI were presented as mean ± SD. Categorical variables like sex and anxiety levels in smokers, non-smokers and healthy patients were expressed in actual numbers and percentages. PPD, CAL, PI, GI and BMI were correlated with anxiety levels in smokers, non-smokers and healthy groups by performing one-way ANOVA. Post hoc multiple comparisons were made by using Bonferroni multiple comparison test. Categorical variables were compared by performing Pearson’s chi-squared test. For small numbers Fischer’s Exact test was used. Correlation between the number of cigarettes and bidis (frequency of smoking) and different anxiety levels was assessed by computing Spearman’s rank correlation for non-normalized data. Correlation between duration of smoking and periodontal parameters (PPD and CAL) was assessed by computing Pearson’s correlation for normalized data. P < 0.05 was considered as statistical significance. All data analyses were carried out using a commercially available software program,STATA Version 13.0.

## Results

The clinical parameters of PPD and CAL were assessed for the three groups in three different anxiety levels of mild, moderate and severe. The mean values of PPD in patients with mild, moderate and severe anxiety levels for smokers, non-smokers as well as the healthy groups are presented in Table 1. Intra-group comparison of PPD in smokers with mild, moderate and severe anxiety levels indicated a direct relationship between PPD and anxiety levels. With an increase in anxiety levels, there was an increase in PPD values. These findings were highly significant (P < 0.0001). A similar trend was observed in non-smokers and healthy groups, though these comparisons did not yield statistical significance (Table 1). The parameter of PPD when compared amongst smokers and non-smokers was non-significant in patients with mild and severe anxiety levels (P < 0.001). The comparisons amongst smokers and healthy subjects as well as non-smokers and healthy subjects yielded highly significant results in patients with mild, moderate and severe anxiety levels (Table 2).

**Table 1:** Correlation of anxiety levels with PPD (probing pocket depth) (in mm) and CAL (clinical attachment levels] (in mm) in smokers and non-smokers and healthy subjects

**Anxiety level**	**PPD** **(mean ± SD** ^‡^ **)**			**CAL** **(mean ± SD** ^‡^ **)**		
	**Smokers**	**Non-smokers**	**Healthy**	**Smokers**	**Non-smokers**	**Healthy**
Mild	4.59 ± 0.22	4.63 ± 0.28	1.32 ± 0.19	5.00 ± 0.15	4.88 ± 0.38	-
Moderate	5.46 ± 0.34	4.88 ± 0.29	1.47 ± 0.33	5.70 ± 0.47	5.13 ± 0.48	-
Severe	5.55 ± 0.35	4.87 ± 0.50	1.65 ± 0.24	5.95 ± 0.28	5.25 ± 0.15	-
P-value	<0.0001^*^	0.0607^†^	0.1057^†^	0.6766^†^	0.6467^†^	-

^*^highly significant ^†^non-significant ^‡^SD = Standard deviation

**Table 2:** Multiple Comparisons of mean differences (mean diff) of PPD [Probing Pocket Depth] (in mm) and CAL [Clinical Attachment Levels] (in mm) with anxiety levels in Smokers, Non Smokers and Healthy subjects

**Periodontal** **Parameters**	**Anxiety Levels**								
	**Mild**			**Moderate**			**Severe**		
	**S** ^|^ **Vs NS** ^¶^	**S** ^|^ **Vs H** ^#^	**NS** ^¶^ **Vs H** ^#^	**S** ^|^ **Vs NS** ^¶^	**S** ^|^ **Vs H** ^#^	**NS** ^¶^ **Vs H** ^#^	**S** ^|^ **Vs NS** ^¶^	**S** ^|^ **Vs H** ^#^	**NS** ^¶^ **Vs H** ^#^
	**Mean** **diff** **(P-value)**	**Mean** **diff** **(P-value)**	**Mean** **diff** **(P-value)**	**Mean** **Diff** **(P-value)**	**Mean** **diff** **(P-value)**	**Mean** **diff** **(P-value)**	**Mean** **diff** **(P-value)**	**Mean** **diff** **(P-value)**	**Mean** **diff** **(P-value)**
**PPD**	0.04 (1.00)^†^	3.27 (<0.001)^*^	3.31 (<0.001)^*^	0.58 (<0.001)^*^	3.99 (<0.001)^*^	3.41 (<0.001)^*^	0.68 (0.011)^†^	3.90 (<0.001)^*^	3.22 (<0.001)^*^
	(1.00)^†^	(<0.001)^*^	(<0.001)^*^	(<0.001)^*^	(<0.001)^*^	(<0.001)^*^	(0.011)^†^	(<0.001)^*^	(<0.001)^*^
**CAL**	0.11 (0.878)^†^	5.00 (<0.001)^*^	4.88 (<0.001)^*^	0.57 (0.011)^§^	5.70 (<0.001)^*^	5.13 (<0.001)^*^	0.70 (0.002)^§^	5.95 (<0.001)^*^	5.25 (<0.001)^*^
	(0.878)^†^	(<0.001)^*^	(<0.001)^*^	(0.011)^§^	(<0.001)^*^	(<0.001)^*^	(0.002)^§^	(<0.001)^*^	(<0.001)^*^

^*^highly significant ^†^non significant ^§^P < 0.05, significant ^|^S = Smokers ^¶^NS = Non Smokers ^#^H = Healthy

In both the smoker and the non-smoker groups there was a consistent rise in CAL with an increase in anxiety levels. The mean values of CAL for patients in smoker and non-smoker groups are shown in Table 1. However, when an intra-group comparison was performed, the results were statistically non-significant. The parameter of CAL when compared between smokers and non-smokers was non-significant in patients with a mild anxiety level, was statistically significant in patients with a moderate anxiety level and was significant in patients with a severe anxiety level (P = 0.002). When compared between smokers and healthy as well as non-smokers and healthy, it was highly significant for patients with mild, moderate and severe anxiety levels (Table 3).

**Table 3:** Correlation of anxiety levels with GI (gingival index) and PI (plaque index) in smokers and non-smokers and healthy subjects

**Anxiety Level**	**GI (mean ± SD** ^‡^ **)**			**PI (mean ± SD** ^‡^ **)**		
	**Smokers**	**Non-smokers**	**Healthy**	**Smokers**	**Non-smokers**	**Healthy**
**Mild**	1.46 ± 0.19	2.13 ± 0.21	-	1.97 ± 0.32	2.02 ± 0.17	0.63 ± 0.26
**Moderate**	1.71 ± 0.21	2.66 ± 0.30	-	2.17 ± 0.28	2.33 ± 0.08	0.54 ± 0.28
**Severe**	2.25 ± 0.41	2.84 ± 0.06	-	2.61 ± 0.52	2.68 ± 0.19	0.78 ± 0.20
**P-value**	<0.001^*^	<0.001^*^	-	0.005^§^	<0.001^*^	0.5116^†^

^*^highly significant ^†^non significant ^‡^SD = Standard deviation ^§^P < 0.05, significant

In relation to correlation between anxiety levels and GI, the intra-group GI in smokers with mild (mean GI of 1.46 ± 0.19 mm), moderate (mean GI of 1.71 ± 0.21 mm) and severe (mean GI of 2.25 ± 0.41 mm) anxiety levels showed highly significant differences (P < 0.001). The same trend was seen with the non-smoker group wherein mild (mean GI of 2.13 ± 0.21), moderate (mean GI of 2.66 ± 0.30) and severe (mean GI of 2.84 ± 0.06) anxiety levels exhibited highly significant differences (P < 0.001) on intra-group comparison. There was a consistent increase in the anxiety levels in smokers, non-smokers and the healthy group, respectively. In both the smoker and the non-smoker groups, intra-group comparisons exhibited highly significant differences with P = 0.005 for smokers and P < 0.001 for non-smokers (Table 3).

The mean BMI values for smokers with mild, moderate and severe anxiety levels were 21.67 ± 1.95 kg/m^2^, 24.55 ± 3.62 kg/m^2^ and 28.98 ± 4.25 kg/m^2^, respectively. The intra-group distinctions were highly significant (P = 0.0011). The mean BMI values for non-smokers were 26.74 ± 6.09 kg/m^2^, 29.84 ± 3.32 kg/m^2^ and 33.34 ± 1.64 kg/m^2^ for mild, moderate and severe anxiety levels. The intra-group comparisons for non-smokers and periodontally healthy patients showed statistically non-significant results. In relation to the correlation of anxiety levels and obesity, the intra-group comparisons demonstrated a consistent surge in the percentage of obesity with a rise in the anxiety levels in all the three groups and it was statistically significant except for periodontally healthy patients where it was non-significant (Table 4). In relation to the correlation of BMI and PPD, there was an increase in PPD in obese patients as compared to the non-obese patients in smokers, non-smokers and healthy groups. CAL was also seen to increase in obese patients as compared to non-obese patients in smokers and non-smokers (Table 5). Multiple inter-group comparisons of periodontal parameters and obesity revealed highly significant differences (P < 0.001) (Table 6).

**Table 4:** Correlation of anxiety levels and obesity (expressed as percentage) in smokers and non-smokers and healthy subjects

**Anxiety Levels**	**Obese**			**Non obese**		
	**Smokers**	**Non-smokers**	**Healthy**	**Smokers**	**Non-smokers**	**Healthy**
Mild	0	41.17%	4.7%	100%	58.82%	95.2%
Moderate	14.28%	77.7%	28.57%	85.71%	22.2%	71.42%
Severe	55%	100%	50%	44.4%	0	50%
P-value	0.016^§^	0.042^§^	0.077^†^	0.016^§^	0.05^†^	0.077^†^

^†^non-significant ^§^P < 0.05, significant

**Table 5:** Correlation of obesity with PPD (probing pocket depth) (in mm) and CAL (clinical attachment levels) (in mm) in smokers and non-smokers and healthy subjects

**Obesity**	**PPD (mean ± SD** ^‡^ **)**			
	**Smokers**	**Non-smokers**	**Healthy**	**P-value**
**Non-obese**	5.13 ± 0.46	1.33 ± 0.23	<0.0001^*^
**Obese**	5.78 ± 0.30	4.92± 0.20	1.69 ± 0.23	<0.0001^*^
	CAL (mean ± SD^‡^)			
	Smokers	Non-smokers	Healthy	P-value
**Non-obese**	5.46 ± 0.43	4.70 ± 0.26	-	<0.0001^*^
**Obese**	6.13 ± 0.38	5.21 ± 0.35	-	<0.0001^*^

^*^highly significant ^‡^SD = Standard deviation

**Table 6:** Multiple comparisons of obesity with mean differences of PPD (probing pocket depth) (in mm) and CAL (clinical attachment levels) (in mm) in smokers and non-smokers and healthy subjects

**Periodontal Parameters**	**Obese**						**Non-obese**					
	**S** ^|^ **vs NS** ^¶^		**S** ^|^ **vs H** ^#^		**NS** ^¶^ **vs H** ^#^		**S** ^|^ **vs NS** ^¶^		**S** ^|^ **vs H** ^#^		**NS** ^¶^ **vs H** ^#^	
	**Mean diff**	**P-value**	**Mean diff**	**P-value**	**Mean diff**	**P-value**	**Mean diff**	**P-value**	**Mean diff**	**P-value**	**Mean diff**	**P-value**
**PPD**	0.86	<0.001^*^	>4.09	<0.001^*^	3.23	<0.001^*^	0.67	<0.001^*^	3.80	<0.001^*^	3.12	<0.001^*^
**CAL**	0.91	<0.001^*^	6.13	<0.001^*^	5.21	<0.001^*^	0.75	<0.001^*^	5.46	<0.001^*^	4.70	<0.001^*^

^*^highly significant ^|^S = Smokers ^¶^NS = Non-smokers ^#^H = Healthy

## Discussion

Over the years, an overall acceptable understanding of susceptible traits for chronic periodontitis has been achieved; however, the inconsistent periodontal disease severity still remains unclear. Preliminary reports have suggested a relationship between psychosocial factors and clinical characteristics identified with the severity of the disease.^16,17^ It has been observed in these studies that practical discrepancies exist in terms of uniform criteria to classify periodontal disease, different methodologies, absence of control group and modifying factors, making it inappropriate to draw a concrete conclusion.

The clinical indicators of severity of periodontal disease such as PPD and CAL showed a considerable increase in patients with severe anxiety in both smokers and non-smokers. Both of these parameters exhibited greater severity in smokers than non-smokers. This indicated a dual mechanism of periodontal disease progression and severity associated with smoking and anxiety. The possible explanation of increased periodontal destruction is the deregulation of immune system in smokers and also a tendency to neglect oral hygiene in patients with severe anxiety levels. These findings concur with those reported by Genco et al, 1999, and Dmitrescu and Kawamura, 2010.^17,18^

The results of our study also revealed identical observations for the parameters of GI and PI. However, the GI and PI values were seen to be more pronounced for non-smokers as compared to smokers, also increasing with an increase in anxiety levels. With regards to GI, the expression of gingival inflammation in the non-smoker group could be attributed not only to the high PI scores but also seem to be related to the anxiety states of patients. The expression of gingival inflammation in the smoker group is suppressed due to smoking. Smoking tends to disguise gingival inflammation by constricting blood vessels of the gingiva.^19^ Under such circumstances, in the non-smokers group, due to inflammatory changes within the gingival tissues, there is bound to be more plaque accumulation, which was confirmed by the results of our study. These findings are partly different from those reported by Johannsen et al 2005^20^, wherein periodontal disease and gingival inflammation were observed to be intense in anxious smokers than in non-anxious smokers, irrespective of dental plaque. The authors seemed to have ignored the findings of suppression of gingival inflammation in smokers. In addition, 22 patients in this study were diagnosed with aggressive periodontitis, which in itself exhibits minimal amounts of gingival inflammation. The present study is better suited and designed to comment on this issue, as we have graded the anxiety levels and evaluated the GI scores for smokers as well as non-smokers. In addition, this investigation comprised of patients, all of whom were diagnosed with chronic periodontitis.

The rise in PI scores with increasing anxiety levels is attributed to the fact that with higher anxiety levels amongst patients, there is decreased tendency towards adopting and following proper oral hygiene measures, which ultimately leads to more plaque accumulation with corresponding effect on periodontal tissues. Interestingly, in this study, a direct relationship was observed between obesity and anxiety levels and smoking was found to act as a modifying factor in this relationship.

A positive association has been reported between obesity and periodontal disease.^21^The present study reported a considerable escalation in values of clinical parameters such as PPD and CAL for obese patients as compared to non-obese ones. This finding was similar for smokers as well as non-smokers with a respective increase in values for obese patients. Inter-group comparison of these parameters for obese as well as non-obese patients yielded highly significant results. These findings are similar to those reported by Saito et al^7^, but are somewhat different from those of Dalla Velchia et al,^21^ where the authors found that female non-smokers were 3.4 times more probable to be detected with periodontitis compared to non-smoking females with normal weight, and no notable correlation was found for males. However, in our study with a majority of patients being males it was observed that obese patients exhibited greater levels of PPD and CAL, indicating greater severity of periodontal disease as compared to non-obese patients.

Also we examined the influence of smoking on the association of obesity and periodontal disease by advocating separate analysis for smokers and non-smokers. The results showed a positive association not only for smokers but even for non-smokers. However, the association was significantly greater for smoking patients, perhaps pointing again towards a dual mechanism of pathogenesis, involving impaired immune response, increased risk for infectious diseases and decreased phagocyte activity.

An additional finding in our study revealed that amongst the smokers, mild, moderate and severe anxiety levels were associated with an increased duration of smoking and the number of cigarettes smoked per day. Also it was observed that the longer the duration of smoking, the greater was the intensity of periodontal destruction as manifested by increased PPD and CAL. These findings at the best can be considered to be suggestive of a cumulative effect and indicate a coherent and biologically plausible representation of the association between smoking, anxiety, obesity and periodontal disease.

The present study does have certain confines and limits. The cross sectional study design prohibits drawing definitive conclusions in terms of causality, because it lacks ability to appraise between cause and effect. The HAM-A scale used for psychological measures to assess stress and anxiety sometimes exhibits poor ability to discern differences between anxiolytic and antidepressant effects. The possibility of unknown factors such as attitudinal and behavioral responses affecting the study variables cannot be ruled out.

## Conclusion

There is growing concern regarding the association of anxiety, obesity, smoking and periodontal disease both in smokers and non-smokers. However, the relationship appears to be more pronounced in smokers. It is thus logical to contemplate that social environment can modify psychological traits which in turn may have an impact upon physiological processes leading to disease susceptibility.

## Acknowledgments

The authors would like to acknowledge the staff of the Department of Periodontics and Implantology, VSPM Dental College and Research Centre, Nagpur for their support.

## Authors’ contributions

AK and RK were responsible for the main design and concept of the study. VL and RK carried out the literature search. AK and VL were responsible for performing the statistical analysis and data compilation. AK and RK drafted and edited the manuscript. All the authors have read and approved the final manuscript.

## Funding

The study was self-funded.

## Competing interests

The authors declare no competing interests with regards to the authorship and/or publication of this article.

## Ethics approval

The study was approved by the Institutional Ethics Committee of VSPM Dental College and Research Centre (Code of Ethical Certificate No. VSPM’S DCRC/DEAN/ETHICS COMMITTEE/05/2015).
